# Smoking and fracture risk in men: a meta-analysis of cohort studies, using both frequentist and Bayesian approaches

**DOI:** 10.1038/s41598-022-13356-1

**Published:** 2022-06-03

**Authors:** Yingke Xu, Yueyang Bao, Megan Wang, Qing Wu

**Affiliations:** 1 Department of Epidemiology and Biostatistics, School of Public Health, University of Nevada, Las Vegas, NV 89154 USA; 2 College of Sciences, Nevada Institute of Personalized Medicine, University of Nevada Las Vegas, Las Vegas, NV 89154 USA; 3 Department of Biology, McMaster University, Hamilton, ON L8S 4L8 Canada; 4 Department of Biology and Biological Engineering, California Institute of Technology, Pasadena, CA 91125 USA

**Keywords:** Risk factors, Epidemiology

## Abstract

Past studies indicate that men are more likely to smoke and be at higher risk of smoking-related conditions than women. Our research aimed, through meta-analysis, to assess the association between smoking and fracture risk in men. The following databases were searched, including MEDLINE, EMBASE, Scopus, PsycINFO, ISI Web of Science, Google Scholar, WorldCat, and Open Grey, for identifying related studies. A random-effects model was used to pool the confounder-adjusted relative risk (R.R.). Frequentist and Bayesian hierarchical random-effects models were used for the analysis. The heterogeneity and publication bias were evaluated in this study. Twenty-seven studies met the inclusion criteria. Overall, smoking is associated with a significantly increased risk of fracture in both the frequentist approach (R.R., 1.37; 95% confidence interval: 1.22, 1.53) and the Bayesian approach (R.R., 1.36; 95% credible interval: 1.22, 1.54). Significant heterogeneity was observed in the meta-analysis (Higgin's I^2^ = 83%) and Cochran's Q statistic (*p* < 0.01). A significant association was also observed in multiple pre-specified sensitivity and subgroup analyses. Similar results were observed in the group containing a large sample size (≥ 10,000 participants), and the group has a small sample size (< 10,000 participants); the pooled R.R was 1.23 (95% confidence interval, 1.07–1.41) and 1.56 (95% confidence interval, 1.37–1.78), respectively. With the Bayesian method, the effect size was 1.23 (95% credible interval, 1.05, 1.45) for the large sample size group and 1.57 (95% credible interval, 1.35, 1.82) for the small sample size group. Smoking is associated with a significant increase in fracture risk for men. Thus, smoking cessation would also greatly reduce fracture risk in all smokers, particularly in men.

## Introduction

Osteoporotic fractures are a major cause of morbidity and disability in older people, often leading to their premature death^1^. As the global population ages, osteoporotic fractures are expected to increase significantly in the coming decades^2^. Worldwide, the number of people aged 50 years or older and who were at high risk of osteoporotic fracture was around 158 million in 2010, and that number is expected to double by 2040^3^. In the United States, data from 2013–2014 indicated that around 8.3% of adults should have received osteoporosis treatment because they were at a 20% or greater 10-year risk of major osteoporotic fractures^3^. From 2006 to 2025, annual osteoporotic fracture events and costs for affected populations in the United States are projected to grow by more than 48%^2^. Thus, osteoporotic fracture prevention is essential for both high-risk individuals and for society in general.

Smoking is the single most preventable cause of disease, disability, and death in the United States^4^. Recent data suggested that there are still approximately 34.2 million adult smokers in this country^5^. Specifically, men are more likely to smoke than women in the US since 16.7% of adult males and 13.6% of adult females smoke cigarettes^6^. In addition, smoking accounted for an estimated 3.1 million years of potential life lost for male smokers and 2.0 million years for female smokers during 2000–2004^7^, suggesting that men are at higher risk of smoking-related conditions than women. Prior studies found that smoking was associated with a significantly increased risk of fractures^8,9^. Smoking increases the risk of spine and hip fracture to 32% and 40% in men, respectively^10^. The one-year mortality rates were as high as 20.6%^11^ and 37.1%^12^ among male smokers with spine and hip fractures. Hence, a reliable estimate of the association between smoking and fractures in men is crucial, which might help to improve their recognition of the dangers of smoking.

Although a prior meta-analysis was conducted and found a significant association between smoking and hip fractures in men^13^, that study was completed five years ago. As a result, several large-scale eligible cohort studies published in recent years were not included^14–19^. As well, the prior meta-analysis only focused on hip fracture^13^, while more current related research has well documented that smoking harms overall bone physiology, thus leading to increased fractures in many other skeletal regions^20^. Therefore, a comprehensive and updated meta-analysis about the association between smoking and fractures in men is needed. Therefore, our current meta-analysis aimed to include both frequentist and Bayesian approaches in order to quantify all eligible cohort studies that assessed the association between smoking and fractures in men. With the Bayesian method, we can estimate the probabilities that smoking increases fracture risk by more than 0%, 10%, and 20%, which the frequentist approach is unable to provide. Therefore, using both classical and advanced methodology in our meta-analysis allows us to gather more comprehensive and accurate information about the effects of smoking on fractures among men.

## Methods

This meta-analysis was conducted in accordance with the Meta-Analysis of Observational Studies in Epidemiology (MOOSE) guidelines^21^, with reference to Preferred Reporting Items for Systematic Reviews and Meta-Analysis (PRISMA)^22^. The study objectives, primary outcomes, literature search strategy, inclusion and exclusion criteria, study selection methods, data extraction, and data synthesis were all defined in advance in the meta-analysis research protocol (in Supplementary). We also pre-specified the sensitivity and subgroup analyses that we planned to conduct this meta-analysis in the protocol.

### Data sources and searches

Two investigators (Y.X. and Y.B.) conducted a comprehensive literature search. Electronic searches were conducted on MEDLINE, using the following terms: *men, fractures, osteoporosis, smoking, cigarette,* and *tobacco*, with no restrictions on language, year of publication, or publication status. Using the same strategy, we also conducted literature searches of EMBASE, PsycINFO, SCOPUS, and ISI Web of Science. The above search terms were adapted for other database searches, according to the syntax of each specific database. The last literature search was conducted on April 28, 2021. We also searched Google Scholar, WorldCat Dissertations, and Open Grey. Experienced librarians were consulted to ensure the comprehensiveness of the literature search. Two investigators (M.W. and Y.B.) independently examined reference lists from the original studies and related meta-analyses and reviews.

### Study selection

The following criteria were used to screen relevant references: (1) prospective or retrospective cohort studies designs; (2) reported smoking status (never, ever, or current smoker); (3) had risk estimates for any fracture or provided sufficient information to estimate fracture risk; and (4) reported results for men. At the initial selection stage, the two investigators independently screened each article's title and abstract retrieved from the electronic search. Only those citations that both reviewers deemed irrelevant were excluded. References with a disagreement between the two reviewers were included for a further full review. In the second phase of the study selection, each reference's full content obtained during the screening stage was reviewed and assessed by the investigators independently. For duplicate publications from the same study cohort, we included in our meta-analysis the study with the largest sample size or effect size adjusted for the largest number of confounders. Disagreements or uncertainties were discussed and resolved through adjudication from a third investigator (Y.X.) when needed. We only included studies that reported relative risk (R.R.) or hazard ratio (H.R.) of fracture associated with smoking or those with the necessary data for calculating R.R. in the current meta-analysis. The agreement between investigators was evaluated using the κ statistic, a robust statistic for inter-rater reliability testing.

### Study appraisal

The methodologic quality of each included study was scored independently by the two researchers (M.W. and Y.B.), using the Newcastle–Ottawa Scale^23^. No major disagreements or discrepancies arose between the two investigators; minor differences were resolved by rechecking the original reports and by discussion. As recommended by the MOOSE study group^21^, the quality scores were not used as weights in the meta-analysis. However, quality scores were used in the subgroup analysis (score > 7 versus ≤ 7).

### Data abstraction

The two reviewers (M.W. and Y.B.) performed data extraction independently. Before the study, a standard data abstraction form was developed. The following information was recorded: titles, authors, types of publication (journal article, abstract, or unpublished data), characteristics of study (year of publication, country of origin, inclusion and exclusion criteria, number of participants, number of cases, and duration of follow-up), characteristics of participants (age and race, if applicable), assessment of exposure (smoking), method of ascertainment of outcomes, outcomes (fractures, along with the corresponding regions), and risk estimates (adjusted R.R. and H.R., corresponding 95% confidence intervals, adjustment of confounders, and stratification abstraction). When multiple estimates were presented in the original studies, the estimates with most confounders were adjusted, and the estimate of current smokers was chosen for overall pooled analysis when applicable. Corresponding estimates from the subgroup analyses in the original studies were abstracted when appropriate. One study^24^ did not report adequate data to compute the effect size. We attempted to contact the corresponding author for additional information but were unsuccessful.

### Statistical analysis

The summary measures used in this meta-analysis were confounder-adjusted R.R. or H.R. for fractures. For studies that reported the estimates by subgroups only, the overall effect size was estimated by a meta-analysis of the reported subgroup's estimates. Before we pooled the data, R.R. or H.R. was transformed into their natural logarithms in order to stabilize the variance and normalize the distribution. We derived H.R. or R.R. natural logarithm variance from the corresponding 95% CIs provided in the original reports. Both frequentist and Bayesian hierarchical random-effects models were utilized for the synthesis analysis. In the frequentist meta-analysis, the DerSimonian-Laird method^25^ was used to calculate the pooled R.R. and variance. In the Bayesian meta-analysis, Gaussian distribution with an unknown effect size (θ_i_) and known within-study variance ${\delta }_{i}^{2}$ was assumed for each log R.R. (denoted as φ_i_). The set of θ_i_ across the original studies was also assumed to follow a Gaussian distribution, with an unknown mean (μ) and across-study variance (τ^2^), where μ was the estimate of the overall log R.R. and τ^2^ was a measure of the between-study variation. The prior distribution of τ^2^ was assumed to follow an improper uniform distribution, and the prior distribution for τ^2^ was assumed to be non-informative. The probabilities that current smoking use increases fracture risk by more than 0%, 10%, or 20% were estimated and reported. Heterogeneity was assessed with Cochran's Q statistic and Higgins's index^26,27^. Univariate and multiple meta-regression analysis was performed to explore heterogeneity. Baujat plot was used to identify studies that had high heterogeneity^28^, and the effect size was estimated after removing outlying/influential studies.

Several pre-specified sensitivity analyses were conducted to assess the robustness of our estimates. The effects of current smoking on fracture risk were calculated with different inclusion criteria, including reporting R.R./H.R., using medical records/hospital dataset, using hip fracture as the outcome, using clinical vertebral fracture as the outcome, and studies focusing on people older than 60 years old. Subgroup meta-analysis stratified by characteristics identified study location, length of follow-up, sample size, year of publication, and quality score. We also conducted a cumulative meta-analysis by performing sequential random-effects pooling, beginning with the earliest qualified report. Each subsequent meta-analysis summarized all eligible reports from the preceding years. To demonstrate the effect of adding reports on the pooled effect size, we presented results chronologically in a forest plot.

A funnel plot created by plotting R.R.s against their standard errors was utilized to examine the potential for publication bias. We also used the Egger test to examine the significance of publication bias. Furthermore, the trim-and-fill method was employed to estimate and adjust unpublished studies' potential effects on the estimated effect size. We used R statistical software (Version 4.0, Core Team, Vienna, Austria) for the data analysis. A p-value of 0.05 or less was considered statistically significant.

## Results

### Literature search

**Figure 1:**
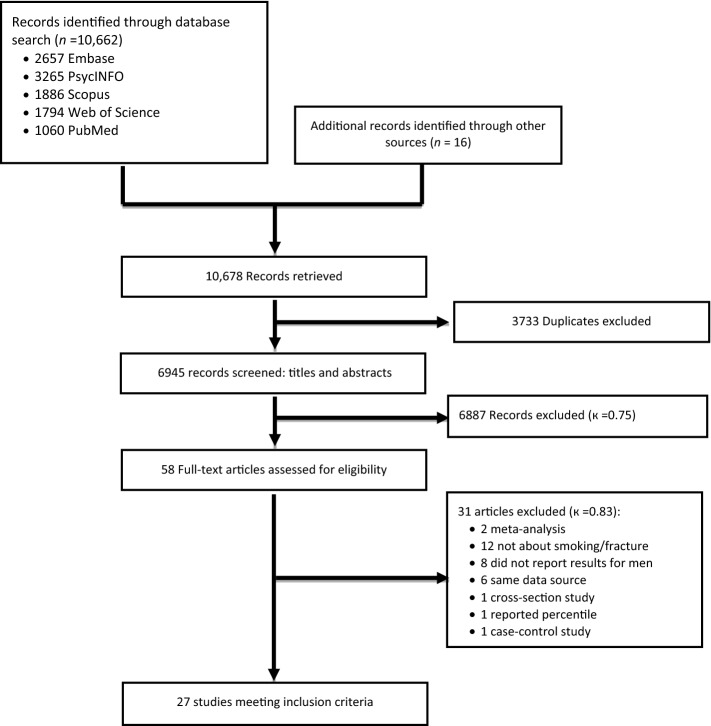
Study Selection for Meta-analysis.

The study flow diagram is illustrated in Fig. 1. After removing duplicate references from different databases, we found a total of 6945 potential references. After investigators Y.B. and M.W. screened titles and abstracts of all these references, 58 full-text research articles were retrieved and assessed for eligibility. The agreement between the two investigators was modest at this initial screening stage (κ = 0.75). After reviewing all full-text articles, twenty-eight studies with fracture data met the inclusion criteria. However, two study reports from the same study team used the same data source but focused on different outcomes^29,30^. Therefore, we combined the two studies as one, and twenty-seven studies were included in the current meta-analysis. Three original studies^31–33^ by Drs. Nguyen et al.^31^, Felsenberg et al.^33^, and De Laet et al.^32^, included in a meta-analysis conducted by Dr. Kanis et al.^34^, also met our inclusion criteria. However, the three studies were updated by Drs. Nguyen et al.^35^, Roy et al.^36^, and van der Klift et al.^37^ with larger study samples, respectively. Thus we included the three corresponding updated studies^35–37^ in the meta-analysis. The agreement between the two investigators was good at this second stage (κ = 0.83). All included studies were published in English.

### Study characteristics

The characteristics of the 27 included studies are summarized in Table 1^8,14–19,29,30,35–53^. For these included studies, the median follow-up time was 9 years, with a range of 2.7–30 years, and the median sample size was 6582, with a range from 258 to 1,174,232. Except for two studies^17,52^, the other twenty-five studies reported using R.R./H.R. However, one of the 25 studies reported H.R. using current smokers rather than non-smokers as the reference^16^. Therefore, we used non-smokers as the reference group and calculated the R.R. based on the provided data for the three mentioned studies^16,17,52^. The potential confounding effects of age were adjusted in 21 studies, and these studies also reported effect size with multiple risk factors adjusted^8,14,19,29,30,36–51,53^. Twenty-one studies were conducted outside of North and South America^8,14–16,18,19,29,30,35–38,40–42,44–46,48,49,51,52^. Thirteen studies only focused on the association between current smoking and hip fractures^8,14,19,29,30,38–41,45,47,49,52,53^. The quality score of the 27 included studies ranged from 4 to 9.

**Table 1:** Characteristics of twenty-three studies examining the association between smoking status and fracture risk.

Author, Year of Publication, Country	Number of Participants, age range of participants	Number of Cases	Mean Follow-Up, Years	Outcome measures	Outcomes	Study Quality Score*	Variables Controlled
Meyer et al., 1993, Norway^8^	27,015 men, aged 35–49 years	128	10.9	Computerized list or manual register	Hip fracture	8	Age, height, BMI, physical activity, diabetes, cerebral stroke, disability pension, marital status
Hemenway et al., 1994, Norway^29,30^	51,529 men aged 40–75 years	338	6	Medical records	Wrist and hip fracture	7	Hip: age, height, BMI, alcohol consumption; wrist: age, alcohol consumption, relative weight, and handedness
Mussolino et al., 1998, U.S.^39^	2879 white men aged 45–74 years	71	13.9	Hospital records	Hip fracture	8	Age, Alcohol Consumption, Chronic Condition(s), BMI, Calories, Protein Quartiles, Weight Loss, Phalangeal Bone Density, Previous Fracture(s) Other Than Hip, Low Nonrecreational Physical Activity, Calcium Intake
Forsén et al., 1998, Norway^38^	14,428 men aged ≥ 20	95	3	Not specified spec	Hip fracture	7	Age, Subjective Health, BMI, Physical Inactivity
Høidrup et al., 2000, Denmark^40^	17,379 men aged ≥ 20 years	447	5–13	Hospital records	Hip fracture	8	Age, Study of Origin, BMI, Alcohol Intake, Physical Activity, School Education
Nguyen et al., 2001, Australia^35^	739 men aged ≥ 60 years	35	7.3	Radiologists' reports	Proximal humerus, forearm, and wrist fracture	7	NA
Roy et al., 2003, Europe^36^	3173 men aged 50–79 years	67	3.8	Radiologists' reports	Vertebral fracture	7	Age, Center of Recruitment
Van der Klift et al., 2004, Netherlands^37^	1377 men aged ≥ 55 years	44	6.3	Radiologists' reports	Vertebral fracture	7	Age, Lumbar Spine BMD, Presence of a Prevalent Vertebral Fracture, History of Any Nonvertebral Fracture at or After Age 50 Years, Smoking Habits
Olofsson et al., 2005, Sweden^41^	2322 men aged 49–51 years	272	30	Radiologists’ reports	Any fracture and hip fracture	8	BMI, Age at First Investigation, Cardiovascular Disease, Diabetes mellitus., Marital Status, Socioeconomic Class, Physical Activity at Work, Leisure Time Physical Activity, Alcohol Consumption
Holmberg et al., 2006, Sweden^42^	22,444 men aged 27–61 years	2422	16	Hospital records	Fragility fracture	**7**	Age, BMI, resting pulse, diabetes, serum triglycerides, serum cholesterol, γ-glutamyl transferase, serum creatinine, poor self-rated health
White et al., 2006, US^43^	5101 men aged ≥ 44 years	501	20	Hospital records	Hip, wrist, and spine fracture	7	Hip: Age at entry, Previous fracture, Glaucoma, No. of children, Attitude; Wrist: Age at entry, Previous fracture, Glaucoma, Rheumatoid arthritis, High blood pressure; Spine: Age at entry, Previous fracture, Alcohol, High blood pressure, Attitude
Moayyeri et al., 2009, U.K.^46^	11,476 men aged 40–79 years	276	11.3	Health Authority database	Any fracture and hip fracture	8	Age, History of fracture, BMI, Alcohol intake
Koh et al., 2009, Singapore^45^	27,913 men aged 45–74 years	276	7.1	Hospital database	Hip fracture	8	Age at Recruitment, Year of Recruitment, Dialect Group, Level of Education, Weekly Vigorous Work or Strenuous Sports, BMI
Hippisley-Cox et al., 2009, U.K.^44^	1,174,232 men aged 30–85 years	7934	6.8	Computerized records	Osteoporotic Fracture and hip fracture	9	Age, BMI, Smoking Status, Alcohol Consumption, Rheumatoid Arthritis, Cardiovascular Disease, Type 2 Diabetes, Asthma, Current Tricyclic Antidepressants, Current Corticosteroids, History of Falls, Liver Disease
Stolee et al., 2009, Canada^47^	13,773 men aged ≥ 65 years	223	2.7	Health information system	Hip fracture	6	Age, Osteoporosis, Parkinson's disease, ADL decline, Uses ambulation aide,
Trimpou et al., 2010, Sweden^49^	7495 men aged 46–56 years	451	30	Hospital diagnosis	Hip fracture	8	Age, Height, BMI, Physical activity, Coffee consumption, Alcoholic intemperance, Stroke before fracture, Dementia before fracture
Jutberger et al., 2010, Sweden^48^	3003 men aged 69–80 years	209	3.3	Computerized X-ray archives	Any fracture	8	Age, Center, Physical Activity, Calcium Intake, Weight, Height, Cancer, COPD, Stroke, Myocardial Infarction, DM, Glucocorticoid Treatment
Ma et al., 2011, US^50^	8006 men aged 45–68 years	513	5	Questionnaire	Hip, spine, and forearm fracture	6	Age, Education, BMI, Grip strength, Upper arm girth, Standing height, Alcohol, Dietary calcium, physical activity index, Glucose, Diabetic medication, Coffee, Milk
Øyen et al., 2014, Norway^52^	2147 men aged 46–74 years	56	9.8	Hospital records	Hip fracture	5	NA
Cauley et al., 2016, U.S.^53^	5994 men aged > 65 years	178	8.6	Medical records	Hip fracture	7	Age, Race, Site, Femoral Neck BMD
Lobo et al., 2017, Spain^14^	1976 men aged ≥ 55 years	50	16	Hospital records	Hip fracture	8	Age, coupled, Illiterate, Alcohol, Weight, Depression, Dementia, Basic activity of daily living
Alhambra et al., 2020, Sweden^15^	40,112 men aged ≥ 18 years	3974	16.9	Hospital records	All fractures (except face, skull, digits), major osteoporotic fractures, and major traumatic fractures (shaft of humerus, forearm, femur, or lower leg)	5	Weight, Height, Parental Education, Alcohol Consumption
Cho IY et al., 2020, Korea^16^	156,379 men aged ≥ 40 years	9790	10	Hospital records	Lumbar fractures, hip fractures, other fractures, all fractures	6	NA
Preyer O, et al., 2021, Austrian^19^	35, 908 men aged ≥ 50 years	590	18.9	Hospital records	Hip fractures	8	Age at baseline examination, BMI, systolic and diastolic blood pressure, triglycerides, cholesterol, malignant disease, diabetes
Hadaegh F, et al., 2021^18^	3477 men aged ≥ 50 years	151	15.9	Hospital records	Any fracture	5	NA
Domiciano, D.S., et al., 2021^17^	258 men	7	4.3	questionnaire	Non-vertebral fractures	6	NA

*Abbreviations* *BMI* Body mass index, *BMD* Bone mineral density, *COPD* Chronic obstructive pulmonary disease.

*The Newcastle–Ottawa Scale was used for quality score.

### Meta-analysis

**Figure 2:**
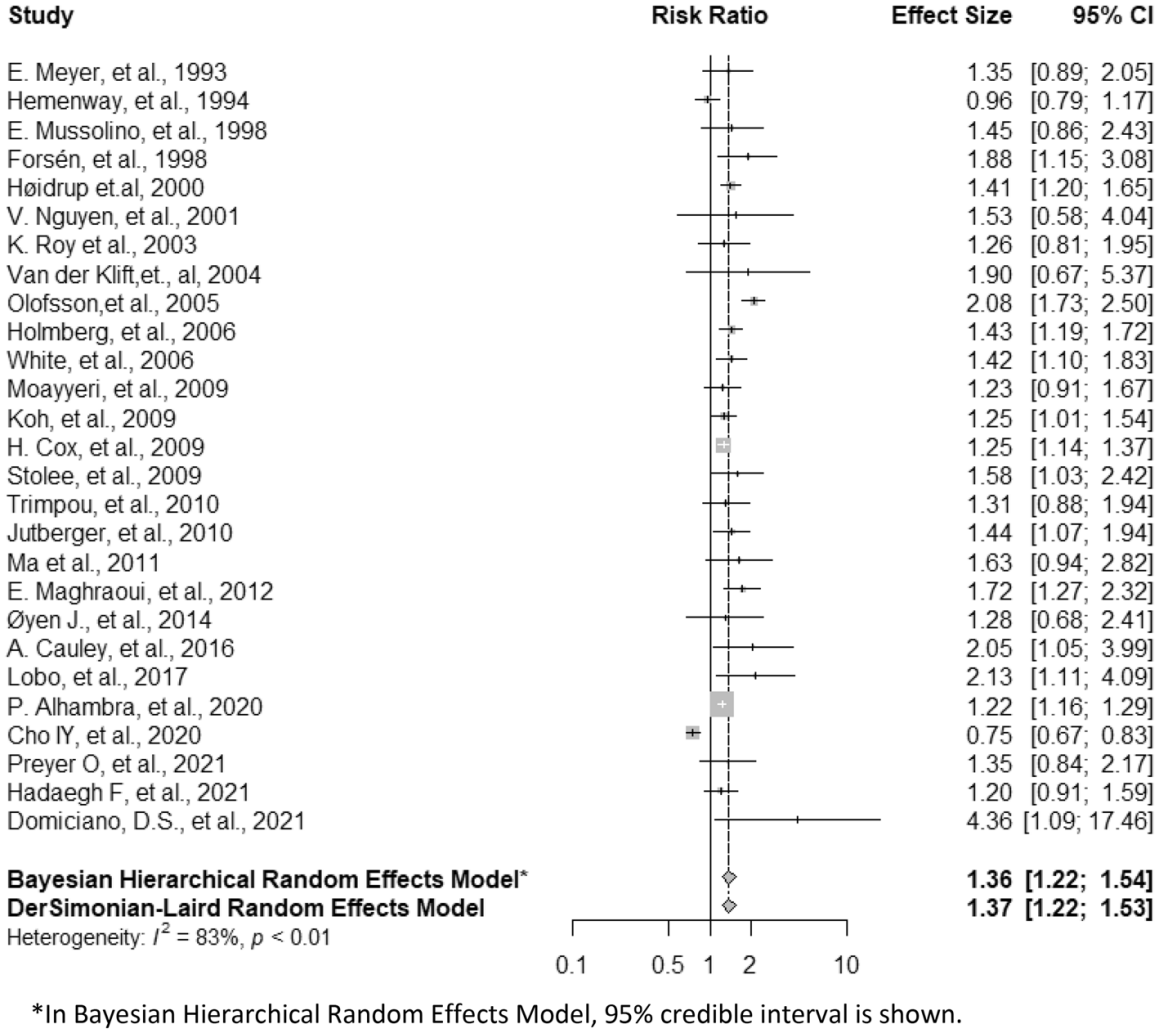
Effects of smoking on the risk of fracture combined, and all eligible studies combined by using frequentist and Bayesian approaches (CI, confidence interval). *In Bayesian Hierarchical Random Effects Model, 95% credible interval is shown.

**Figure 3:**
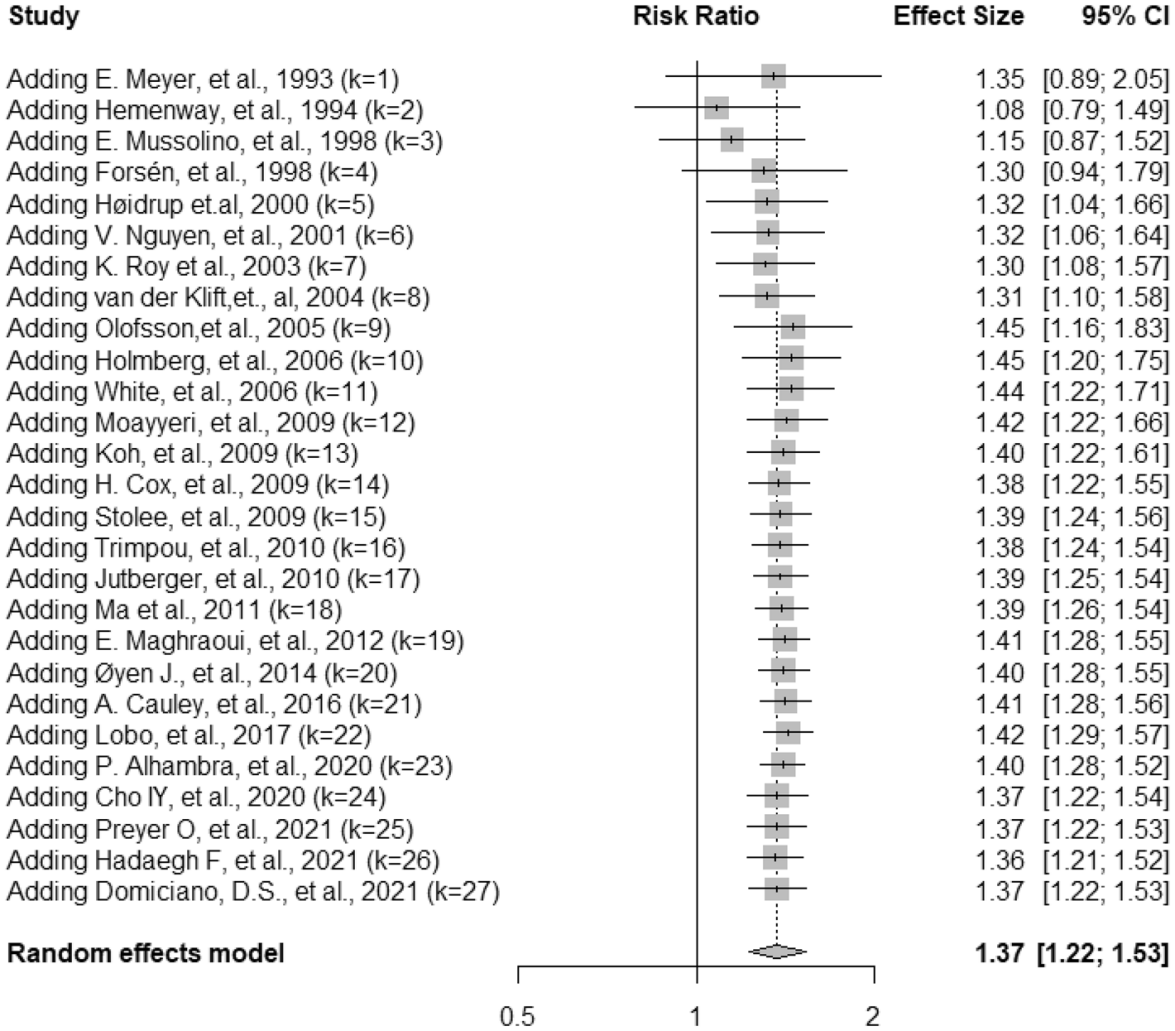
Cumulative random-effects meta-analysis (DerSimonian-Laird method) of smoking on the risk of fracture.

Figure 2 shows the pooled R.R. and corresponding 95% confidence interval (CI) based on the frequentist method and the pooled R.R. with corresponding 95% credible interval (CrI) based on Bayesian approaches. Compared to non-smokers, smokers (including former and current smokers) had an overall R.R. of 1.37 (95% CI, 1.22–1.53) in the frequentist approach and 1.36 (95% CrI, 1.22–1.54) in the Bayesian method. The results from the Bayesian hierarchical random-effects model suggest that the probabilities that smoking increased fracture risk by more than 0%, 10%, and 20% were 99%, 99%, and 98%, respectively. Significant heterogeneity was observed among the twenty-seven studies in this meta-analysis, as the Cochran Q statistic was significant (*p* < 0.01), and the Higgins I^2^ index was 83%. The pooled estimates varied slightly but remained significant when they included studies with different eligibility criteria for the analysis (Table 2). Similar effect size was observed among studies that reported R.R./H.R., and the estimate was 1.38 (95% CI, 1.27–1.50). The effect size slightly increased when studies used hip fracture (R.R., 1.46; 95% CI, 1.24–1.72) or vertebral fracture (R.R., 1.48; 95% CI, 1.28–1.72) as the outcome. When we restricted the analysis to the eight studies that included only participants over 60 years of age, the overall R.R. increased to 1.48 (95%CI, 1.27–1.72). The cumulative meta-analysis further demonstrated an association between current smoking and fracture risk (see Fig. 3). By sequentially accumulating studies according to their publication year, the pooled estimates fluctuated during 1993–2009, and the estimate remained stable afterward.

**Table 2:** Risk of fracture associated with smoking in studies with different inclusion criteria.

Studies included	No. of reports	R.R. (95% CI)	Heterogeneity		
			Q	*P*-value	I^2^, %
All studies	27	1.37 (1.22,1.53)	148.75	< 0.0001	83
Studies reported R.R./H.R	24	1.38 (1.27, 1.50)	54.46	0.0002	57.8
Studies with medical record/hospital database	24	1.33 (1.20, 1.49)	141.37	< 0.0001	83.7
Studies using hip fracture as outcome	13	1.46 (1.24, 1.72)	37.05	0.0002	67.6
Studies included participants 60 + years only	8	1.48 (1.27, 1.73)	4.22	0.75	0
Studies using vertebral fracture as outcomes	4	1.48(1.28, 1.72)	1.83	0.61	0

The frequentist approach and random-effect model were used for analysis unless noted otherwise.

### Subgroup analysis

Table 3 summarizes the effects of smoking on fracture risk in the subgroup analyses. The fracture risk was slightly higher among studies conducted in North/South America (R.R., 1.54; 95% CI, 1.29–1.84) than studies completed in Europe and other regions. We also found studies with follow-ups of more than five years had a lower R.R. (1.31; 95% CI, 1.15–1.50) than studies with less than five follow-up years (R.R., 1.49; 95% CI, 1.33–1.67). Notably, the group with a large sample size (≥ 10,000 participants) had a significantly lower R.R. (1.23; 95% CI, 1.07–1.41) compared to the group with a small sample size (< 10,000 participants) (R.R., 1.56; 95% CI, 1.37–1.78); the between-group difference was significant (*p* = 0.01). Moreover, studies published after 2010 had a similar R.R. (1.39; 95% CI, 1.25–1.54) to those published before or in 2010 (1.37; 95% CI, 1.08–1.75). Except for the sample size group, no significant between-group difference was observed in the subgroup analyses. The estimated effect sizes in the subgroup with the Bayesian method are shown in Supplementary Table 1, all of which were quite similar to the results from the frequentist, which indicated that heterogeneity remained high in most subgroup analyses. In the univariate meta-regression, the results of R^2^ indicate that two variables, location and reported R.R./H.R., could explain the, 31.34% and 58.61% of the heterogeneity, respectively. There was no multicollinearity between the two mentioned variables, and the heterogeneity in this study was further assessed with multiple meta-regression. After adjusting the two variables and their interaction, the significant heterogeneity among included studies was still observed (I^2^, 58.7%; *p*-value = 0.0006). The Baujat plot was then used to detect outlier/influential studies, and two^16,41^ were identified (Supplementary Fig. 1). After excluding the two studies, the pooled effect size for the remaining 25 studies was 1.33 (95%CI, 1.24–1.42) in the frequentist approach, and the I^2^ was 23.4%, while the p-value for the heterogeneity test was 0.14 (Supplementary Fig. 2).

**Table 3:** Stratified analyses of the risk ratio of fracture associated with smoking, by subgroups.

Subgroup	No. of studies	R.R. (95% CI)	Q Statistic	*P*-value for Heterogeneity	I^2^ Value, %	Between-group *p*-value
**Location**						
North/South America	6	1.54 (1.29, 1.84)	3.37	0.64	0	0.37
Europe	16	1.37 (1.24, 1.51)	46.80	< 0.0001	67.9	
Other	5	1.19 (0.82, 1.72)	43.57	< 0.0001	90.8	
**Length of follow-up**						
< 5 years	7	1.49 (1.33, 1.67)	5.18	0.52	0	0.15
≥ 5 years	20	1.31 (1.15, 1.50)	130.24	< 0.0001	85.4	
**Sample size**						
< 10,000	15	1.56 (1.37, 1.78)	19.6	0.14	28.6	0.01
≥ 10,000	12	1.23 (1.07, 1.41)	92.58	< 0.0001	88.1	
**Year of publication**						
≤ 2010	17	1.39 (1.25, 1.54)	39.76	< 0.0001	59.8	0.94
> 2010	10	1.37 (1.08–1.75)	86.17	< 0.0001	89.6	
**Quality score**						
≤ 7	16	1.33 (1.13, 1.56)	101.01	< 0.0001	85.2	0.51
> 7	11	1.43 (1.26, 1.62)	26.88	0.0027	62.8	
**Defined smoking status into three levels (non-smoker, former smoker, and current smoker)**						
Yes	11	1.27 (1.11, 1.46)	14.06	0.17	28.9	< 0.0001
No	16	1.39 (1.21, 1.61)	134.70	< 0.0001	88.9	

The frequentist approach and random-effect model were used for analysis unless noted otherwise.

### Publication bias

**Figure 4:**
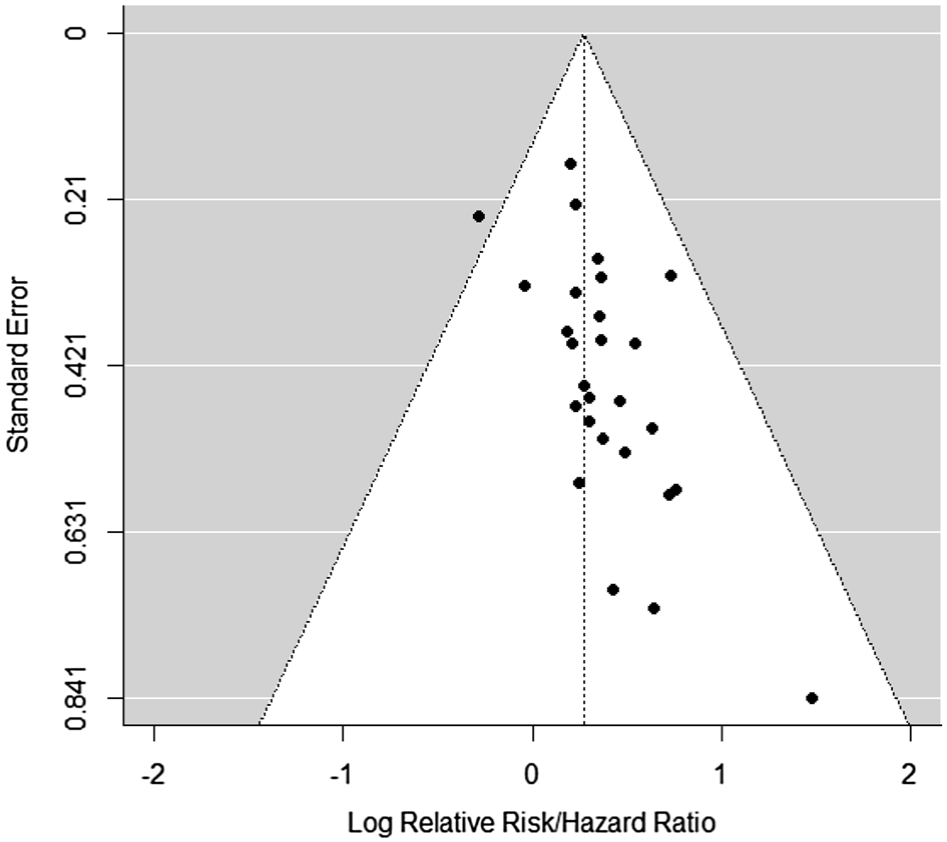
Funnel plot for the detection of publication bias. Data were analyzed using the frequentist meta-analysis approach.

Publication bias was examined by plotting the log R.R.s between smokers and non-users against their standard errors for each study (Fig. 4). Visual inspection of the funnel plot indicated that publication bias might be present. The Egger test (*p* = 0.0024) also indicated significant publication bias in our current meta-analysis. Hence, we employed the trim-and-fill correction to adjust for the publication bias. However, the overall effect size remained significant after the correction (R.R., 1.20; 95% CI, 1.06–1.35).

## Discussion

This meta-analysis summarizes 27 cohort studies to assess the association between smoking and fracture risk among men. Both the frequentist method (R.R., 1.37; 95% CI, 1.22, 1.53) and the Bayesian method (R.R., 1.36; 95% CrI, 1.22, 1.54) showed a significant association between smoking and increased fracture risk. The association between smoking and fracture risk was consistent in all sensitivity analyses with different inclusion criteria, various subgroup analyses, and analysis after excluding two outlier/influential studies, which suggests consistency and robustness of findings in this meta-analysis. Furthermore, although the cumulative meta-analysis showed that the pooled estimate fluctuated during 1993–2009, the study also showed a consistent and significant association between smoking and an increased risk of fractures since 2010. This finding suggests that the addition of future studies would have a limited impact on the overall estimate. Additionally, the 95% CIs were increasingly narrower when studies were organized chronologically, which further demonstrates the robustness of our results.

Our meta-analysis results are consistent with the previous meta-analysis conducted by Dr. Wu et al., that smoking increases the risk of hip fracture in men^13^. However, the previous meta-analysis was published five years ago and thus was unable to integrate findings from recently published, more extensive studies^14–19,54–57^. Moreover, the previous meta-analysis only assessed smoking and hip fracture association. Therefore, the association between smoking and other, more specific fracture outcomes remains unknown. However, we updated the meta-analysis by including the most recent qualified study reports in the present study. We also quantified the association between smoking and overall fracture, along with vertebral fractures. In addition, we included two crucial updated study reports in the present meta-analysis, both with large sample sizes. Dr. Forsen and colleagues published an eligible study in 1994^58^, which was included in the previous meta-analysis. The same research group published another updated report with the same data in 1998; the corresponding updates were included in the present meta-analysis. We also replaced the original study by Paganini-Hill et al.^59^ in the previous meta-analysis, with an updated report by White et al.^43^, in the present meta-analysis. Although both study reports used the same data source, the newer version was more comprehensive. It assessed the association between smoking and hip, wrist, and spine fracture, while the older one only focused on hip fracture, so the current study included the updated findings. Compared to the previous meta-analysis, we employed both the frequentist and Bayesian approaches to evaluate the association between smoking and fracture risk. The results from the two methods were consistent in our study. In addition, the Bayesian meta-analysis provided the probabilities that smoking increases fracture risk by 10% and 20%; such results help male smokers to recognize that smoking is linked to elevated fracture risk. Our findings are also consistent with a prior meta-analysis by Dr. Kanis and his colleagues^34^ that current male smokers had a significantly higher fracture risk than non-current smokers. Compared to Dr. Kanis and colleagues' meta-analysis, our meta-analysis not only included all additional recent eligible studies but also replaced three original studies by Drs. Nguyen et al.^31^, Felsenberg et al.^33^, and De Laet et al.^32^ in Dr. Kanis's meta-analysis with the corresponding updated studies by Drs. Nguyen et al.^35^, Roy et al.^36^, and van der Klift et al.^37^, respectively. The three updated studies had a larger sample size, which might contribute to a more precise estimate because the variance and standard error decrease as the sample size increaseds^60^. Thus, our meta-analysis is likely to yield a more accurate estimate of the effect size.

The underlying mechanism of how smoking influences fracture risk is not fully understood. One potential reason could be the decreased bone mineral density (BMD) caused by smoking^61^. Low BMD is the primary cause of osteoporotic fracture risk and is a measure widely used in clinical practice to identify patients at an increased risk of fracture^56^. The biological plausibility of BMD loss due to smoking can be linked to the effects of nicotine and cadmium in cigarette smoke on bone cells^61^. In addition, smoking is associated with decreased vitamin D levels. People with low vitamin D are more likely to have low BMD and are at a higher risk of suffering a fracture^62^. On the other hand, smoking is also associated with reducing calcium absorption^54^, also leading to increased fracture risk. Another potential reason is that smoking has been considered a risk factor for injury^57^, which is linked to fractures. A study in elderly persons found a 28% increase in smokers' accidental injury over non-smokers, and smoking and nicotine are inhibitory factors in wound and fracture healing^55^. Smoking also interferes with tissue repair processes, leaving tissue more susceptible to injury and fracture^55^.

Our study has several limitations. First, two studies with self-reported data and one without specification about the outcome measures were included. The data from the three studies might be less reliable compared to other data derived directly from medical records. After removing the three studies, the effect size of smoking on fracture risk decreased slightly. Second, due to the different questionnaire designs from the included studies, we could not examine the dose–response relationship between smoking and the risk of fractures. Third, publication bias is suspected in the current meta-analysis, as indicated by the funnel plot and Egger test. However, the pooled estimate remained significant after we adjusted for publication bias by using the trim-and-fill method. Finally, the adjustment for confounders in all the included articles varies, which may exaggerate or underestimate the findings. Nevertheless, this limitation unlikely altered our meta-analyses conclusion; the consistent findings from sensitivity and subgroup analyses suggested that our current study findings are reliable and robust.

## Conclusion

In summary, our comprehensive meta-analysis found a significant association between smoking and increased risk of fractures. Our findings were consistent in both frequentist and Bayesian approaches, as well as all subgroup analyses, sensitivity analysis, and the analysis with publication bias correction. More importantly, our results have crucial implications in public health, with the most apparent being that quitting smoking can reduce an individual's risk of bone fracture, both now and later in life.

## Supplementary Information

Supplementary Information.
