# Supplementary material for: Smoking and fracture risk in men: a meta-analysis of cohort studies, using both frequentist and Bayesian approaches
Source: Sci Rep. 2022 Jun 3;12:9270. doi: 10.1038/s41598-022-13356-1 (PMC9166727; doi:10.1038/s41598-022-13356-1)
Supplement: Supplementary file 1 — Supplementary Information. [file 41598_2022_13356_MOESM1_ESM.docx]

**
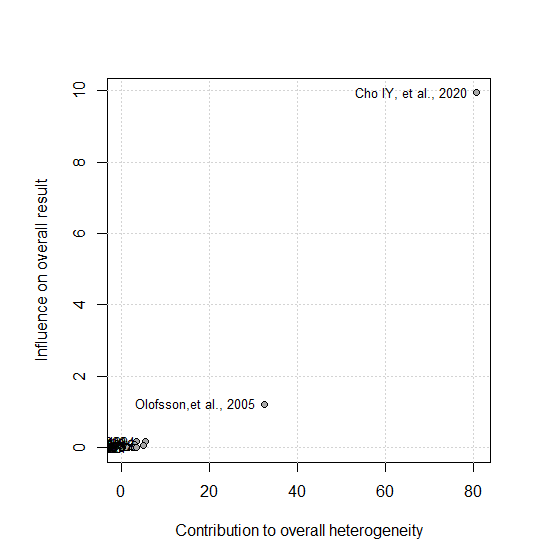
Supplementary Figure 1** Baujat plot for identifying outlier/influential studies.

**
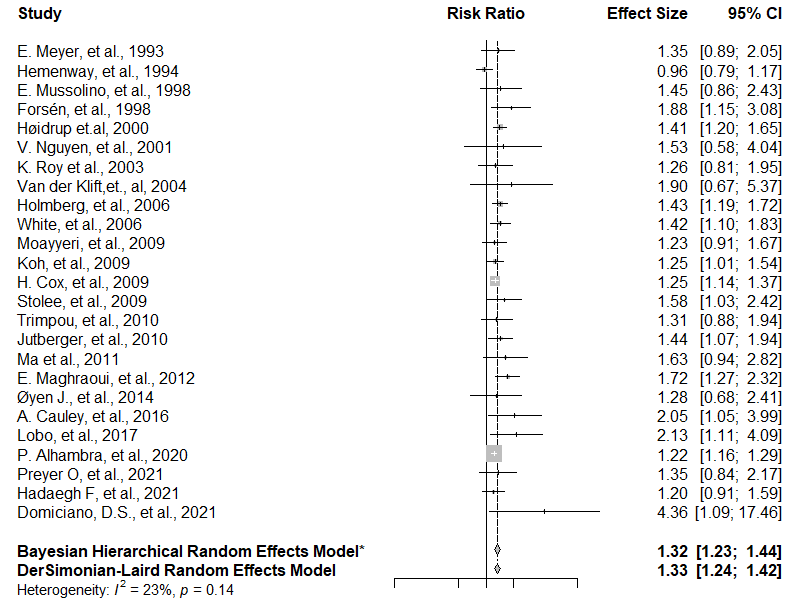
Supplementary Figure 2** Effects of smoking on the risk of fracture combined, and 25 studies combined by using frequentist and Bayesian approaches (CI, confidence interval;).

*In Bayesian Hierarchical Random Effects Model, 95% credible interval is shown.

**
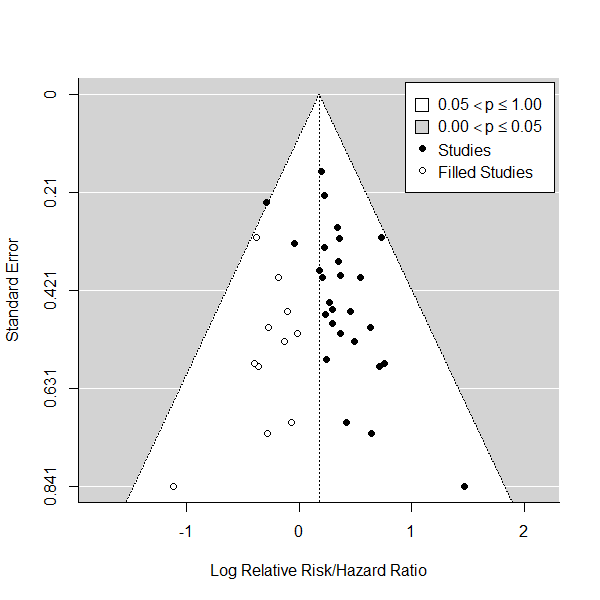
Figure 3** Funnel plot with trim and fill. Data were analyzed using the frequentist meta-analysis approach.

**Supplementary Table 1.** Stratified analyses of the risk ratio of fracture associated with smoking by subgroups.

| **Subgroup** | **No. of studies** | **R.R. (95% CI)** | **Q Statistic** | **P-value for Heterogeneity** |
| --- | --- | --- | --- | --- |
| Location |  | | | |
| North/South America | 6 | 1.58 (1.20,2.18) | 3.37 | 0.36 |
| Europe | 16 | 1.38 (1.21, 1.57) | 46.80 | <0.0001 |
| Other | 5 | 1.19 (0.68, 2.17) | 43.57 | <0.0001 |
| Length of follow-up |  | | | |
| <5 years | 7 | 1.52 (1.27, 1.85) | 5.18 | 0.8 |
| ≥5 years | 20 | 1.31 (1.14, 1.52) | 130.24 | <0.0001 |
| Sample size |  | | | |
| <10,000 | 15 | 1.57 (1.35, 1.82) | 19.60 | 0.2 |
| ≥10,000 | 12 | 1.23 (1.05, 1.45) | 92.58 | <0.0001 |
| Year of publication |  |  |  |  |
| ≤2010 | 17 | 1.39 (1.23, 1.46) | 39.76 | <0.0001 |
| >2010 | 10 | 1.38 (1.03, 1.91) | 86.17 | <0.0001 |
| Quality score |  | | | |
| ≤7 | 16 | 1.33 (1.12, 1.62) | 101.01 | <0.0001 |
| >7 | 11 | 1.43 (1.23, 1.66) | 26.81 | <0.0001 |
| Defined smoking status into three levels (non-smoker, former smoker, and current smoker) |  | | | |
| Yes | 11 | 1.28 (1.09, 1.55) | 14.06 | 0.1 |
| No | 16 | 1.40 (1.19,1.65) | 134.70 | <0.0001 |

**Protocol of Meta-analysis on the Smoking and fracture risk**

**Objectives**:

Primary Objective: The study focuses on analyzing the association between smoking and fracture risk found in the frequentist method.

Secondary Objective: Bayesian method will be used to estimate the effect size of smoking on fracture risk

**Background and significance**:

Osteoporotic fractures are a major cause of morbidity and disability in older people, often leading to their premature death ^1^. In the United States, data from 2013-2014 indicated that around 8.3% of adults should have received osteoporosis treatment because they were at a 20% or greater 10-year risk of major osteoporotic fractures^2^. From 2006 to 2025, annual osteoporotic fracture events and costs for affected populations in the United States are projected to grow by more than 48%^3^. Thus, osteoporotic fracture prevention is essential for both high-risk individuals and society.

Smoking is the single most preventable cause of disease, disability, and death in the United States^4^. In the US, men are more likely to smoke than women since 16.7% of adult males and 13.6% of adult females smoke cigarettes^5^. Previous studies found that smoking was associated with a significantly increased risk of fractures^6,7^. A valid estimate of the association between smoking and fractures in men is crucial as this awareness might help to improve their recognition of the dangers of smoking.

A meta-analysis was conducted five years ago and found a significant association between smoking and hip fractures in men^8^. Thus, several large-scale eligible cohort studies published in recent years were not included^9-14^. Meanwhile, the mentioned meta-analysis only focused on hip fracture^8^, while related research has well documented that smoking harms overall bone physiology, leading to increased fractures in many other skeletal regions^15^. Therefore, a comprehensive and updated meta-analysis about the association between smoking and fractures among men is needed. Our current meta-analysis aimed to use both frequentist and Bayesian approaches to quantify all eligible cohort studies that assessed the association between current smoking and fractures in men.

**Methods**:

**Inclusion Criteria**:

Language restricted in the English and Chinese;

Conducted in human subjects;

Prospective cohort study or retrospective cohort study;

Fracture as an outcome;

Smoking as the exposure;

Relative risk or hazard ratio and variance (or raw data) reported

**Search strategy**:

We will conduct electronic searches in MEDLINE, EMBASE, Scopus, Cochrane Database, and Google Scholar in order to identify related published articles, employing the following Medical PubMed Search Syntax: 1. (smoking OR cigarette OR tobacco) 2. (fracture OR hip fracture OR wrist fracture OR vertebral fracture) 3. (men OR male) 4. #1 AND #2 AND #3. The above search terms will be adapted for other database searches according to the syntax of each specific database

**Study Selection:**

Two independent reviewers will review the title and abstract from the searched results for further scrutiny and document the number of articles with specific data retrieved from each database. According to the inclusion criteria, reviewers will keep the related articles and record the basic information. If there is any doubt, they will retrieve and review the full text and consult with a third reviewer. For the excluded articles, reviewers will need to record the specific reasons for exclusion and the number of excluded references. After this, reviewers will need to check the included articles, remove duplicate studies, and document the number of included studies. Finally, the level of agreement in the study selection between the two reviewers should be calculated with kappa in each stage.

**Study Quality Assessment**:

Because the quality of included studies has a critical impact on the meta-analysis, we should evaluate each study with a proper assessment tool and record the information. In this study, we will analyze the cohort study; thereafter, we will use The Newcastle-Ottawa Scale to evaluate the quality of each included study.

**Data Extraction**

Data will be extracted by the two independent investigators from the eligible studies using the standard form. Disagreement will be discussed, and additional reviewers will help to solve the problem if necessary. A collection form will be utilized for the information, such as the name of authors, year of publication, study design, characteristics of participants (sample size, distribution of age, race, and so on), fracture or not.

**Analysis Plan**

Before aggregating data from studies, factors such as differences in patient population, study location, follow-up, and outcome measurement need to be considered. The summary measures used in this meta-analysis were confounder-adjusted R.R. or H.R. for fractures. For studies that reported the estimates by subgroups only, the overall effect size will be estimated by a meta-analysis of the reported subgroup’s estimates. Before we pool the data, R.R. or H.R. will be transformed into their natural logarithms in order to stabilize the variance and normalize the distribution. We derived the H.R. or R.R.’s natural logarithm variance from the corresponding 95% C.I.s provided in the original reports. Both frequentist and Bayesian hierarchical random-effects models will be utilized for the synthesis analysis. The meta-analysis will be done by random effect processes. The *Q* statistic and I^2^ will be reported within the forest plot. The Q test provides information about the presence of heterogeneity but does not offer a quantitative evaluation of the extent of this heterogeneity. The I^2^ offers the advantage of providing a quantitative assessment of the heterogeneity in effect sizes that are due to between-study variability.

Heterogeneity among reviewed manuscripts can be initially observed in the original graphic output and 95% confidence intervals (CIs). We will perform several subgroup analyses. Subgroups will be stratified by characteristics identified study location, length of follow-up, sample size, year of publication, and quality score.

**Reference**

1 Colón-Emeric, C. S. & Saag, K. G. Osteoporotic fractures in older adults. *Best Pract Res Clin Rheumatol* **20**, 695-706, doi:10.1016/j.berh.2006.04.004 (2006).

2 Odén, A., McCloskey, E. V., Kanis, J. A., Harvey, N. C. & Johansson, H. Burden of high fracture probability worldwide: secular increases 2010-2040. *Osteoporosis international : a journal established as result of cooperation between the European Foundation for Osteoporosis and the National Osteoporosis Foundation of the USA* **26**, 2243-2248, doi:10.1007/s00198-015-3154-6 (2015).

3 Burge, R. *et al.* Incidence and Economic Burden of Osteoporosis-Related Fractures in the United States, 2005–2025. *J Bone Miner Res* **22**, 465-475, doi:<https://doi.org/10.1359/jbmr.061113> (2007).

4 Centers for Disease, C. *et al.* in *How Tobacco Smoke Causes Disease: The Biology and Behavioral Basis for Smoking-Attributable Disease: A Report of the Surgeon General* (Centers for Disease Control and Prevention (US), 2010).

5 Jamal, A. *et al.* Current Cigarette Smoking Among Adults - United States, 2005-2015. *MMWR. Morbidity and mortality weekly report* **65**, 1205-1211, doi:10.15585/mmwr.mm6544a2 (2016).

6 Meyer, H. E., Tverdal, A. & Falch, J. A. Risk factors for hip fracture in middle-aged Norwegian women and men. *Am J Epidemiol* **137**, 1203-1211, doi:10.1093/oxfordjournals.aje.a116622 (1993).

7 Murray, R. The role of smoking in the progressive decline of the body’s major systems. (Public Health England, 2014).

8 Wu, Z.-J., Zhao, P., Liu, B. & Yuan, Z.-C. Effect of Cigarette Smoking on Risk of Hip Fracture in Men: A Meta-Analysis of 14 Prospective Cohort Studies. *PloS one* **11**, e0168990-e0168990, doi:10.1371/journal.pone.0168990 (2016).

9 Lobo, E. *et al.* Gender differences in the incidence of and risk factors for hip fracture: A 16-year longitudinal study in a southern European population. *Maturitas* **97**, 38-43, doi:10.1016/j.maturitas.2016.12.009 (2017).

10 Prieto-Alhambra, D. *et al.* Smoking and Alcohol Intake but Not Muscle Strength in Young Men Increase Fracture Risk at Middle Age: A Cohort Study Linked to the Swedish National Patient Registry. *J Bone Miner Res* **35**, 498-504, doi:10.1002/jbmr.3917 (2020).

11 Cho, I. Y. *et al.* Effects of smoking habit change on hospitalized fractures: a retrospective cohort study in a male population. *Archives of osteoporosis* **15**, 29, doi:10.1007/s11657-020-0686-y (2020).

12 Domiciano, D. S. *et al.* Incidence and risk factors for osteoporotic non-vertebral fracture in low-income community-dwelling elderly: a population-based prospective cohort study in Brazil. The São Paulo Ageing and Health (SPAH) study. *Osteoporosis international : a journal established as result of cooperation between the European Foundation for Osteoporosis and the National Osteoporosis Foundation of the USA* **32**, 747-757, doi:10.1007/s00198-020-05669-6 (2021).

13 Hadaegh, F. *et al.* Sex-specific incidence rates and risk factors for fracture: A 16-year follow-up from the Tehran lipid and glucose study. *Bone* **146**, 115869, doi:10.1016/j.bone.2021.115869 (2021).

14 Preyer, O. *et al.* Serum uric acid is associated with incident hip fractures in women and men - Results from a large Austrian population-based cohort study. *Maturitas*, doi:10.1016/j.maturitas.2021.03.005 (2021).

15 Kanis, J. A., Johnell, O., Oden, A., Johansson, H. & McCloskey, E. FRAX and the assessment of fracture probability in men and women from the UK. *Osteoporosis international : a journal established as result of cooperation between the European Foundation for Osteoporosis and the National Osteoporosis Foundation of the USA* **19**, 385-397, doi:10.1007/s00198-007-0543-5 (2008).
